# Toxic Effect of Acute Cadmium and Lead Exposure in Rat Blood, Liver, and Kidney

**DOI:** 10.3390/ijerph16020274

**Published:** 2019-01-18

**Authors:** Milena Andjelkovic, Aleksandra Buha Djordjevic, Evica Antonijevic, Biljana Antonijevic, Momcilo Stanic, Jelena Kotur-Stevuljevic, Vesna Spasojevic-Kalimanovska, Milos Jovanovic, Novica Boricic, David Wallace, Zorica Bulat

**Affiliations:** 1 Health center Kosovska Mitrovica, 38220 Kosovska Mitrovica, Serbia; millena.andjelkovic@gmail.com (M.A.); momcilostanickm@gmail.com (M.S.); 2 Department of Toxicology “Akademik Danilo Soldatović”, Faculty of Pharmacy, University of Belgrade, 11221 Belgrade, Serbia; evicaa@pharmacy.bg.ac.rs (E.A.); abiljana@pharmacy.bg.ac.rs (B.A.); zorica.bulat@pharmacy.bg.ac.rs (Z.B.); 3 Department of Medical Biochemistry, Faculty of Pharmacy, University of Belgrade, 11221 Belgrade, Serbia; jkotur@pharmacy.bg.ac.rs (J.K.-S.); vkalima@pharmacy.bg.ac.rs (V.S.-K.); 4 Institute of Physiology and Biochemistry, Faculty of Biology, University of Belgrade, 11221 Belgrade, Serbia; jovanovic@bio.bg.ac.rs; 5 Institute of Pathology, Faculty of Medicine, University of Belgrade, 11221 Belgrade, Serbia; novica.boricic@med.bg.ac.rs; 6 Department of Pharmacology and Toxicology, School of Biomedical Science, Oklahoma State University Center for Health Sciences, Tulsa, OK 741071898, USA; david.wallace@okstate.edu

**Keywords:** cadmium, lead, mixture, oxidative stress, toxicity, rats

## Abstract

*Background*: Cadmium and lead are widespread and non-biodegradable pollutants of great concern to human health. In real life scenarios, we are exposed to mixtures of chemicals rather than single chemicals, and it is therefore of paramount importance to assess their toxicity. In this study, we investigated the toxicity of Cd and Pb alone and as a mixture in an animal model of acute exposure. *Methods*: Experimental groups received a single treatment of aqueous solution of Cd-chloride (15 and 30 mg/kg body weight (b.w.) and Pb-acetate (150 mg/kg b.w.), while the mixture group received 15 mg Cd/kg b.w. and 150 mg Pb/kg b.w. Toxic effects of individual metals and their mixture were investigated on hematological and biochemical parameters, and the redox status in the plasma, liver, and kidneys of treated Wistar rats. *Results*: Tissue-specific changes were recorded in various parameters of oxidative damage, while the accumulation of metals in tissues accompanied the disturbances of both hematological and biochemical parameters. It was observed that the level of toxic metals in tissues had a different distribution pattern after mixture and single exposure. *Conclusions*: Comprehensive observations suggest that exposure to Cd and Pb mixtures produces more pronounced effects compared to the response observed after exposure to single metal solutions. However, further research is needed to confirm toxicokinetic or toxicodynamic interactions between these two toxic metals in the organisms.

## 1. Introduction

Cadmium (Cd) and lead (Pb) are ubiquitous and non-biodegradable pollutants representing a great concern to human health. Both metals are naturally distributed, but industrial development has dramatically increased their concentrations in the environment [1,2,3]. Industries associated with smelting and mining, manufacturing of batteries, pigments, and ceramic are well-known emitters of Cd and Pb. Increased emissions of both metals in the environment and their non-biodegradability have increased the risk of human exposure. The main routes of Cd and Pb exposure are ingestion and inhalation due to their presence in food, air, and tobacco leaves [1,2,3,4]. The World Health Organization (WHO) has published a list of 10 chemicals or groups of chemicals of concern for human health, which includes Cd and Pb [5]. Additionally, the US Agency for Toxic Substances and Disease Registry (ATSDR) ranked Cd in seventh and Pb in second place on the priority list of dangerous substances [6].

Many in vivo and in vitro studies have been conducted to determine the exact mechanisms of toxicity of Cd and Pb. The present body of knowledge suggests oxidative stress as one of the critical mechanisms of toxicity of both metals, even though neither of these metals is a Fenton’s metal [7,8,9,10]. Other possible mechanisms of toxicity are binding to oxygen, nitrogen, and sulphur ligands, which may affect numerous enzymes and proteins [7,11]; interaction with bioelements [12,13,14]; inhibition of apoptosis [15]; and changes in DNA structure and the inhibition of damaged DNA repair, which may lead to aberrant gene expression [16,17,18].

After absorption, Cd and Pb are distributed in the organisms via red blood cells or proteins [19,20]. A major amount of Cd in red blood cells is bound to high-molecular-weight proteins, while a minor amount is bound to hemoglobin [19]. However, when Pb enters the cell, most of it is bound to hemoglobin rather than the membrane of red blood cells [21]. The hematopoietic system is one of the most sensitive systems and blood represents not only the mode of transportation, but also the critical toxicity target of Cd and Pb [21,22]. Both metals may lead to anemia by various mechanisms [2,10,23]. Cadmium and Pb are transported to the liver, in which they can cause damage and disturbed function. Liver damage can be confirmed by histopathological findings and is often accompanied by increased blood enzyme levels and reduced protein synthesis [24,25,26,27,28]. Toxic effects on kidneys are represented through the structure damage of kidneys and changes in the excretory function [24,25,28,29]. In addition to the metal-toxicity observed in the hematopoietic system, liver, and kidneys, metals have been implicated in various other organ toxicity. Cadmium has been shown to exert toxicity in pancreas, endocrine, cardiovascular, immune, and reproductive systems [1,3,30,31,32], while Pb toxicity has been linked to toxic effects on the nervous, cardiovascular, and reproductive systems [2,10,21,33]. The majority of the Pb body burden is in mineralizing tissues (bones and teeth) [34]. The fact that Cd and Pb have a comparable radius to Ca ions means that both toxic metals can lead to bone damage by displacing Ca ions [11,35]. The International Agency for Research on Cancer (IARC) has classified Cd as carcinogenic to humans (Group 1), while inorganic Pb has been classified as probably carcinogenic to humans (Group 2A) based on limited evidence in humans and sufficient evidence in animals [21,22,36].

Humans are exposed to mixtures of chemicals rather than an individual chemical, and therefore, it is important to establish whether chemical mixtures produce a more pronounced effect compared to individual chemicals. The importance of the evaluation of ”cocktail effects“ has been summarized in the European Commission statement, which highlighted that even low-level exposure to a complex cocktail of pollutants over decades could have a significant effect on the health status of European citizens [37]. The co-exposure to Cd and Pb may implicate possible synergism or antagonism, additive, or new effects that are not observed for single metal exposure [7,38]. A sub-chronic oral toxicity study with different Cd and Pb doses showed that the main target organs were the blood, liver, and kidneys [24]. Fifteen days following the intraperitoneal administration (i.p.) of a Cd and Pb mixture, Pillai et al. [39] reported that Cd was the more reactive of the two metals, while Masso et al. [40] suggested a possible antagonistic effect between Cd and Pb. Clearly, the interactions between the two metals in a combined mixture are complex and warrant further investigation.

We decided to investigate the toxicity of a Cd and Pb mixture in an animal model of acute exposure. The effects of the exposure to single metals and their mixture were investigated on hematological and biochemical parameters and redox status in the plasma, liver, and kidneys of treated rats. Furthermore, the distribution of metals and bioelements was investigated in selected tissues, as well as their histopathology.

## 2. Materials and Methods

### 2.1. Chemicals

All reagents and chemicals were of analytical grade quality or higher purity. Cadmium chloride (CdCl_2_xH_2_O, Merck, Germany) and lead acetate (Pb(CH_3_COO)_2_x3H_2_O, Centrohem, Serbia) were used for oral administration solutions. Standard solutions of Cd, Pb, copper (Cu), and zinc (Zn) (Merck, Germany) were used to create calibration curves for toxic metals and bioelements analysis, while cHNO_3_ (65%, Merck, Germany) and H_2_O_2_ (30%, Sigma-Aldrich, Germany) were used for tissue mineralization. All chemical and reagents for the examination of antioxidant status were purchased from Sigma-Aldrich Chemie (Germany).

### 2.2. Animals

Male Wistar rats were purchased from the Military Medical Academy (Belgrade, Serbia). The experiment was performed on male albino Wistar rats weighing approximately 250 g. Animals were housed under standard controlled conditions (temperature 25 ± 3 °C, relative humidity of 35% to 60%, 12-h light-dark cycle) and allowed free access to standard rat chow and drinking water during the experiment. All experimental procedures were approved by the Ethical Committee on Animal Experimentation of the University of Belgrade, Faculty of Pharmacy (Serbia, project code number III 46009).

### 2.3. Study Design and Experimental Procedure

Following two weeks of acclimatization, rats were randomly divided into five groups: one control group and four experimental groups. Experimental groups that received a single dose of Cd and Pb were composed of six animals, while the number of rats in the Cd and Pb mixture group was seven. The control group was composed of eight animals. Experimental groups received a single treatment of aqueous solution of CdCl_2_ and/or Pb(CH_3_COOH)_2_ in doses: 15 mg Cd/kg body weight (b.w.) (Cd_15_ group), 30 mg Cd/kg b.w. (Cd_30_ group), 150 mg Pb/kg b.w. (Pb_150_ group), and 15 mg Cd/kg b.w. and 150 mg Pb/kg b.w. (Cd_15_ + Pb_150_ group). The control group was treated with water only. The selection of experimental doses was based on our previous research, as well as literature data [24,41,42]. Also, a lower dose of Cd was selected for the mixture treatment in accordance with the results of our previous research [42]. The treatment of all animals was performed by an oral gavage in a volume of 1 mL/kg b.w. Animals were sacrificed 24 h after treatment under light anesthesia.

### 2.4. Tissue Preparations

Blood samples were collected by cardiac puncture through the diaphragm, after anesthetic administration. One aliquot of blood with anticoagulant (heparin) was used for the measurement of hematological parameters, and a second aliquot was wet digested for toxic metals and bioelements measurement. The remaining amount of blood with anticoagulant (heparin) was used to obtain plasma, while the rest of the blood was collected in test tubes without anticoagulant for obtaining serum. Plasma and serum were separated and frozen (−80 °C for plasma and −20 °C for serum) for redox status and biochemical assays, respectively. Organ systems examined, the liver and kidneys, were removed and separated into three parts. One tissue sample was immediately frozen in liquid nitrogen and stored at −80 °C for the investigation of antioxidant status, a second was stored at −20 °C for toxic metals and bioelements analysis, and a third tissue sample was preserved in formalin for histopathological examination.

### 2.5. Toxic Metals and Bioelements Analysis

Heparin blood (approximately 1 mL) and wet tissue samples weighing about 500 mg were placed in Teflon containers with 7 mL cHNO_3_ and 1 mL H_2_O_2_ and mineralized (Milestone START D, SK-10T, Milestone Srl, Sorisole, Italy). Digestion was carried out according to the Milestone’s recommendations. The amount of Cd, Pb, Cu, and Zn in the blood and tissue samples was determined by atomic absorption spectrophotometry (AAS GTA 120 graphite tube atomizer, 200 series AA, Agilent Technologies, Santa Clara, CA, USA). Matrix modifier, 10% Triton X, and 0.5% NH_4_H_2_PO_4_ have been used throughout the whole experimental assay. The accuracy of AAS analyses was validated with standard reference material (SRM) whole blood Level 2 (Seronorm^TM^, Sero, Billingstad, Norway) for blood analyses and with SRM 1577c - Bovine liver (LGS Standard, UK) for tissue samples.

### 2.6. Hematology Analysis

Hematological parameters were measured by the CELL-DYN Ruby analyzer (Abbott, Abbott Park, IL, USA). Multi-angle polarized scatters separation was used for white blood cell and dual angle optical analysis for platelets count. The following hematological parameters were examined: white blood cell count (WBC) with WBC differential count (neutrophils, eosinophils, lymphocytes, basophils, and monocytes), red blood cell count (RBC), hemoglobin concentration (HGB), hematocrit (HCT), mean corpuscular volume (MCV), mean corpuscular hemoglobin (MCH), mean corpuscular hemoglobin concentration (MCHC), and platelet count (PLT).

### 2.7. Biochemical Assays

The measured biochemical parameters in rat serum included blood urea nitrogen (BUN), creatinine (CRE), uric acid (UA), total serum proteins (TP), albumin (ALB), direct bilirubin (DB), total bilirubin (TB), aspartate aminotransferase (AST), alanine aminotransferase (ALT), alkaline phosphatase (ALP), amylase (AMY), lactate dehydrogenase (LDH), iron (Fe), calcium (Ca), inorganic phosphorus (P), magnesium (Mg), and chloride (Cl). All biochemical assays were performed with commercial reagents and according to good laboratory practices on the Beckman Coulter analyzer (AU 480, Beckman Coulter, Brea, CA, USA). Calibrators of tests were traceable to the National Institute of Standards and Technology (NIST) SRM and Beckman Coulter Master Calibrators.

### 2.8. Redox Status Analysis

Tissues samples (liver and kidneys) for redox status assessment were rapidly excised, washed in ice-cold 0.9% NaCl, and homogenized in nine volumes of buffer (0.1 mol/L phosphate buffer, pH 7.4) [43]. Homogenization was carried out by the T10 basic Ultra-Turrax homogenizer (IKA, Staufen, Germany). A portion of homogenate was used for the determination of malondialdehyde (MDA), while the rest was centrifuged for 10 min (at 4 °C) at 800 g and then for 20 min at 9500 g to get post-mitochondrial supernatant (PMS). Assessment of the following parameters was performed: MDA, advanced oxidation protein products level (AOPP), total thiol (SH) groups level, prooxidative-antioxidative balance (PAB), total antioxidative status (TAS), total oxidative status (TOS), oxidative stress index (OSI), and superoxide dismutase activity (SOD) at ILAB 300 plus analyzer (Instrumentation Laboratory, Milan, Italy) and Cary 60 UV-VIS spectrophotometer (Agilent Technologies, Santa Clara, CA, USA).

The concentration of MDA was determined as a thiobarbituric acid-reactive substance (TBARS) by a spectrophotometric assay based on the absorption maximum of the malondialdehyde complex and other TBARS with thiobarbituric acid at 535 nm [44]. The principle of the method for determining AOPP is a two-step measurement of sample absorption in the wavelength range of 200–400 nm, with a characteristic peak at 340 nm. The obtained difference in the measured absorbance values of 340 nm indicates the AOPP value for the given sample [45]. The principle of the method for determining the total SH-groups is as follows: aliphatic thiol compounds in the base environment are reacted with DTNB (2,2’-dinitro-5,5’-dithio-benzoic acid), wherein one mol of thiol produces one mol of *p*-nitrophenol. The resulting color has an absorption peak at 412 nm [46]. Alamandari et al. [47] have developed a method for the determination of PAB using 3,3’,5,5’-tetramethylbenzidine as a chromogen. The method principle for TAS assessment is as follows: the colorless reduced form of ABTS is oxidized to dark green color ABTS^+^ with hydrogen peroxide in an acidic medium (acetate buffer 30 mM, pH 3.6) [48]. The TOS method is based on oxidants present in the sample to oxidize the ferrous ion from the *o*-dianisidine complex to ferric ion. The build-up intensity is directly proportional to the concentration of oxidants present in the sample, with an absorption peak at 560 nm [49]. Oxidative stress degree was calculated as the ratio between TOS and TAS [50]. The method of determining the activity of superoxide dismutase enzymes (SOD, EC 1.15.1.1.) was established in 1971 by Misra and Fridovich [51]. The method is based on the ability of SOD to inhibit the spontaneous autoxidation of adrenaline at pH 10.2. Protein levels in tissues were determined by the Bradford method [52] using bovine albumin as the standard, while plasma protein levels were determined by the standard biuret method [53].

### 2.9. Histopathological Analysis

The preserved tissues (liver and kidneys) were subjected to histopathological examination. Microscopic examinations on paraffin embedded 5 µm tissue sections with hematoxylin-eosin were performed. Each section was examined under an optical microscope.

### 2.10. Statistical Analysis

Statistical analysis was performed using SPSS 18.0 (SPSS Inc. Chicago, IL, USA) software. One-way ANOVA and Kruskal-Wallis nonparametric tests were used. These statistical tests were followed by LSD or the Mann-Whitney *U* test, respectively. *p*-Values less than 0.05 were considered significant.

## 3. Results

### 3.1. Cadmium and Lead Concentration in Tissues

Experimental groups treated with a single dose of Cd had a statistically higher blood concentration of Cd compared to values in the control group. Blood concentrations of Cd in the Cd-Pb mixture group were not different from control values. Both experimental groups receiving a single dose of Cd (15 or 30 mg/kg) demonstrated significantly higher levels of Cd in the liver compared to the control group (*p* < 0.001), while the Cd_30_ group had a higher concentration of Cd in the liver compared to the Cd_15_ group (*p* < 0.01). The measured level of liver Cd in the experimental group receiving Cd and Pb mixture exhibited a statistically significant difference when compared to the control, while difference from the Cd_15_ group was not observed. Additionally, both Cd groups (Cd_15_ group and Cd_30_ group) showed statistically significant higher levels of Cd in kidneys compared to the control group (*p* < 0.001). Experimental groups treated with 30 mg Cd/kg b.w. had a higher concentration of Cd in the kidneys compared to 15 mg Cd/kg b.w. (*p* < 0.05). After mixture treatment, the Cd concentration in the kidneys showed a statistically significant difference when compared to the control and Cd_15_ group (*p* < 0.001, *p* < 0.01, respectively).

**Figure 1:**
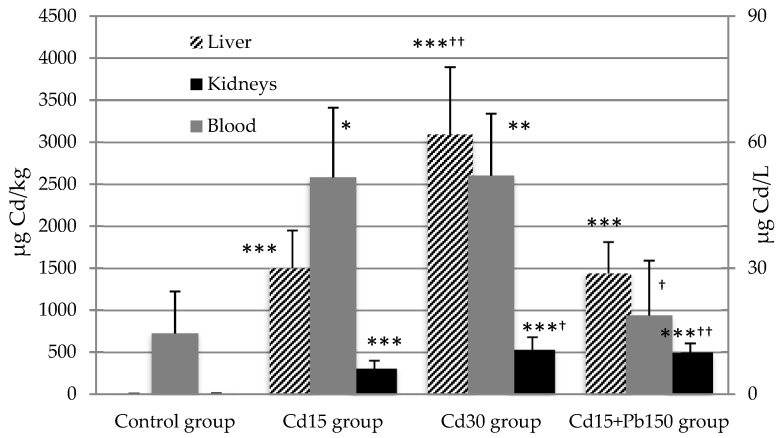
Cadmium concentration in rat blood, liver, and kidneys after acute exposure to Cd and Pb. Cadmium levels in blood are expressed as µg Cd/L and as µg Cd/kg (wet tissues) in liver and kidneys. Values are presented as means and S.D. Statistically significant differences (*p* < 0.05) compared to control group are indicated by *, ^†^ Cd_15_ group. Statistical evaluation was performed using one-way ANOVA followed by LSD post-hoc test for pairwise comparison. * ^†^ *p* < 0.05; ** ^††^ *p* < 0.01; *** ^†††^ *p* < 0.001.

**Figure 2:**
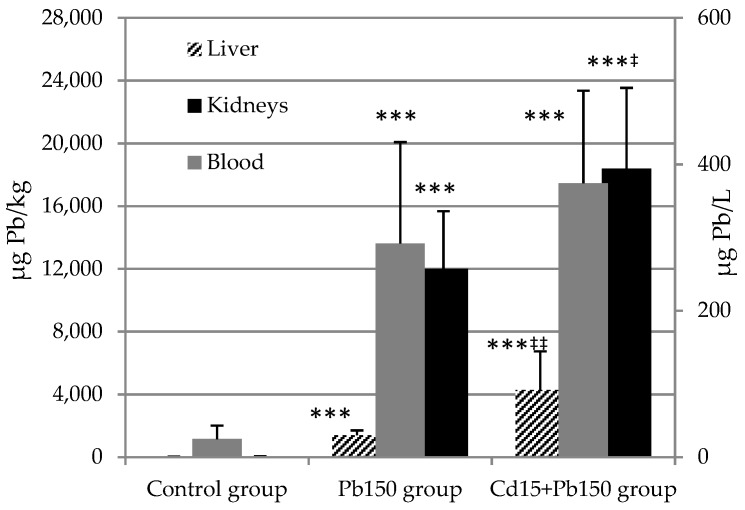
Lead concentration in rat blood, liver, and kidneys after acute exposure to Cd and Pb. Lead levels in blood are expressed as µg Pb/L and as µg Pb/kg (wet tissues) in liver and kidneys. Values are presented as means and S.D. Statistically significant differences (*p* < 0.05) compared to control group are indicated by *, ^‡^ Pb_150_ group. Statistical evaluation was performed using one-way ANOVA followed by LSD post-hoc test for pairwise comparison. * ^‡^ *p* < 0.05; ** ^‡‡^ *p* < 0.01; *** ^‡‡‡^ *p* < 0.001.

Experimental groups that received Pb had higher concentrations of lead in all three of the investigated mediums: blood, liver, and kidneys, compared to values in the control group. Additionally, the Cd_15_ + Pb_150_ group had a statistically higher Pb concentration in the liver and kidneys compared to the Pb_150_ group (*p* < 0.01, *p* < 0.05 respectively), while the difference was not observed in blood. Levels of Cd and Pb in investigated tissues are shown in Figure 1 and Figure 2.

### 3.2. Hematology

Higher doses of Cd produced a significant decrease in the WBC number compared to the control group and to a lower dose of Cd (*p* < 0.001, *p* < 0.01, respectively), while there was no difference in the group receiving the mixture. The change in absolute lymphocyte count had a similar trend, and the Cd_30_ group exhibited a lower count compared to values from the control and Cd_15_ groups (both, *p* < 0.01). A lower dose of Cd also produced a significant decrease in contrast to the control group (*p* < 0.01). Following the administration of Pb, we observed significantly higher absolute neutrophils counts in only the group that received the mixture of toxic metals in contrast to control values (*p* < 0.05), while single Pb treatment did not produce any changes in WBC count.

In all experimental groups, we noticed a decreasing trend in RBC, HGB, and HCT levels compared to values obtained from the control group, yet there was no difference between the treated groups. The most pronounced effects were observed in the Cd_30_ group and Cd_15_ + Pb_150_ group (for all three parameters, *p* < 0.001). Significant changes in MCH were noticed in both Cd-treated groups (Cd_15_ group and Cd_30_ group) when compared to the controls. Neither MCV nor MCHC was affected by the treatment.

Platelet count was significantly increased in the Cd_15_ group (*p* < 0.01), but decreased in the Cd_30_ group, in contrast to control. The Pb given as a single chemical achieved a statistically significant decrease in the PLT count compared to the control group, while Pb given in the mixture only produced a reduction in PLT when compared to the same single dose of Cd (*p* < 0.001). Observed hematological parameters are shown in Table 1.

### 3.3. Biochemical Assays

Acute exposure to the investigated toxic metals administered alone or in mixture form resulted in the altered profile of some biochemical parameters. All experimental groups had lower concentrations of urea compared to the control group, while there was a slight increase in creatinine levels in all experimental groups, only reaching significance in the Cd_30_ group and Pb_150_ group when compared to the controls (*p* < 0.01, *p* < 0.05, respectively). Total proteins and albumin levels were lower after exposure to a mixture relative to the control group (*p* < 0.01, *p* < 0.05, respectively). Additionally, mixture treatment caused a lower proteins level (*p* < 0.05) in contrast to the Pb_150_ group. An elevated level of direct bilirubin was observed in the Pb_150_ group in comparison to the control, while total bilirubin was at least slightly increased in all experimental groups, with only the values from the Cd_15_ group and Cd_30_ group reaching statistical significance compared to the control. Exposure to either toxic metal did not affect the levels of uric acid across all treatment groups.

All experimental groups had lower concentrations of serum iron compared to the control (*p* < 0.001), without any difference among dosing groups. Change in calcium levels was towards reduction, but statistical significance was only achieved in groups that received Pb, with a more pronounced effect in the mixture (*p* < 0.01). A similar trend was noticed for magnesium levels, with reductions not only after Pb administration, but also following the dose of 30 mg Cd/kg b.w. Furthermore, differences within the groups were not observed for either calcium or magnesium. Phosphorus levels were only significantly lower after the administration of 30 mg/kg b.w. Cd when compared to the control (*p* < 0.05). Changes in chloride levels were observed in the group receiving Pb as a single metal and in a group receiving a higher Cd dose when compared to the untreated group, whereas after the application of the mixture, chlorine levels were significantly different, but only for the individual Pb dose group (*p* < 0.01). Observed biochemical parameters are shown in Table 2.

**Figure 3:**
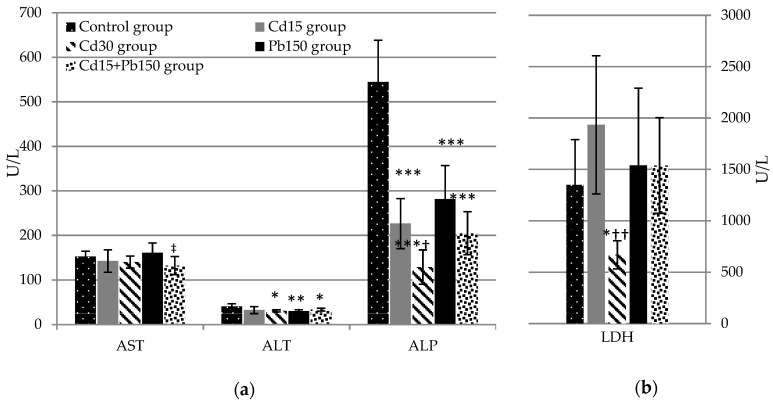
Effect of Cd or/and Pb on serum enzyme activities in rats after acute exposure. Aspartate aminotransferase (AST), alanine aminotransferase (ALT), and alkaline phosphatase (ALP) are represented on panel (**a**), while lactate dehydrogenase (LDH) on panel (**b**). Parameters that were not influenced by Cd and Pb treatment are not listed. Values are presented as means and S.D. Statistically significant differences (*p* < 0.05) compared to control group are indicated by *, ^†^ Cd_15_ group, ^‡^ Pb_150_ group. Statistical evaluation was performed using one-way ANOVA followed by LSD post-hoc test for pairwise comparison. * ^† ‡^ *p* < 0.05; ** ^†† ‡‡^ *p* < 0.01; *** ^††† ‡‡‡^ *p* < 0.001.

All experimental groups exhibited a downward trend in ALT activity, and a statistically significant effect at the higher dose of Cd (*p* < 0.05), as well as in both groups receiving Pb, when compared to the controls. On the other hand, only mixture administration reduced AST activity (*p* < 0.05) compared to Pb given alone. Both Cd doses decreased ALP activity compared to the control group (*p* < 0.001), and higher Cd doses significantly decreased ALP activity (*p* < 0.05) compared to lower Cd doses. Similarly, experimental groups receiving Pb and the mixture showed a decrease in ALP activity relative to the control group (*p* < 0.001). LDH activity was decreased after 30 mg/kg b.w. Cd administration in contrast to the untreated controls and the Cd_15_ group (*p* < 0.05, *p* < 0.01, respectively), while changes in AMY activity were not observed. Investigated enzyme activities in rat serum are shown in Figure 3.

### 3.4. Redox Status

Blood MDA level was increased after the administration of 30 mg/kg b.w. Cd, but only when compared to the lower Cd dose (*p* < 0.05). After exposure to the mixture, both MDA and AOPP blood levels were increased compared to groups receiving single metal. Additionally, mixture treatment induced a significant elevation of blood AOPP compared to control values (*p* < 0.001). We did not observe any toxic metal-induced changes in the overall levels of SH-groups and PAB in blood. Liver AOPP concentration was increased in all experimental groups receiving a single metal, yet we did not observe any differences between these groups. AOPP levels after mixture treatment were similar to control values, but were observed to be lower when compared to a single Cd dose. Other redox parameters, such as MDA, SH-groups, and SOD activity, were not altered in rat liver after acute exposure to toxic metals. The higher dose of Cd produced significant increases in kidney MDA levels when compared to the group treated with a lower Cd dose (*p* < 0.05). Elevated MDA levels were observed in both Pb treatment groups, with more pronounced effects after treatment with the mixture compared to the controls (*p* < 0.001). Furthermore, mixture administration produced a significant elevation in MDA concentration in the kidney compared to a single Cd dose (*p* < 0.01). AOPP levels, SH-groups, and SOD activity remained unchanged in kidneys after toxic metals treatments. Observed redox parameters in rat blood, liver, and kidneys are presented in Table 3.

**Figure 4:**
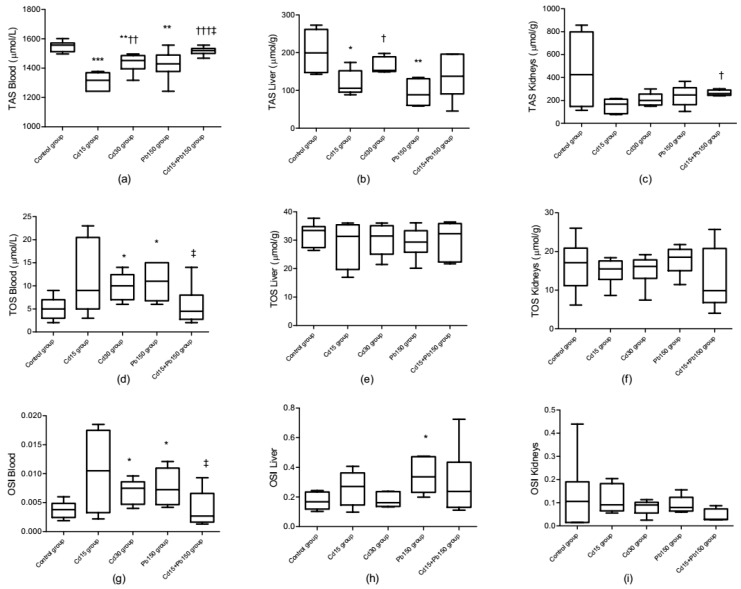
Effect of Cd or/and Pb on total antioxidative status (TAS, panel (**a**–**c**)), total oxidative status (TOS, panel (**d**–**f**)), and oxidative stress index (OSI, panel (**g**–**i**)) values in rat blood, liver, and kidney after acute exposure. Levels of TAS and TOS are expressed on wet tissues. The box represents interquartile range (25–75th percentile), the line within the box represents median value, and ends of the whiskers represent the minimum and maximum values within the group. Statistically significant differences (*p* < 0.05) compared to control group are indicated by *, ^†^ Cd_15_ group, ^‡^ Pb_150_ group. Statistical evaluation was performed using the Kruskal-Wallis nonparametric test followed by Mann-Whitney *U* test for pairwise comparison. * ^† ‡^ *p* < 0.05; ** ^†† ‡‡^ *p* < 0.01; *** ^††† ‡‡‡^ *p* < 0.001.

Total antioxidative status, total oxidative status, and oxidative stress index in rat plasma, liver, and kidneys are presented in Figure 4. Blood TOS levels were increased in all experimental groups exposed to a single metal, but the increase was only significant in the Cd_30_ group and Pb_150_ group compared to control values. TAS content was inversely affected by the type of metal treatment. Groups receiving a single metal had lower blood TAS levels compared to the control, but TAS levels were significantly higher in the mixture group compared to groups treated with a single metal. The degree of oxidative stress in blood was calculated as the ratio between TOS and TAS, which exhibited an upward trend after exposure to individual metals. Only in the highest dose, the Cd_30_ and Pb_150_ groups, did this trend reach statistical significance. The most robust effect in the liver was observed after exposure to a single Pb dose (*p* < 0.01), resulting in a reduction in TAS content compared to the control group. Moreover, the highest liver OSI index value was noticed in the Pb_150_ group, which was in line with the lowered TAS value. No statistically significant changes were observed in kidney TOS and TAS levels compared to the control group, hence the OSI index also remained unchanged.

The obtained redox parameters after acute exposure with Cd or/and Pb were used to calculate three scores: a score of oxidative damage (DS), a score of antioxidative defenses (protection score, PS), and the so-called OXY-score (as a global score of oxidative balance). The protection score was calculated as an average of standardized antioxidant variables by calculating *Z* scores (TAS, SOD, and SH-groups), while the damage score was calculated as an average of standardized prooxidant factors by calculating *Z* scores (TOS, PAB, MDA, and AOPP). The OXY-score was calculated by subtracting the PS from the DS using the equation given by Veglia et al. [54]. The OXY-score is approximately equal to zero when the sum of all the analytes is approximately the average of normal values, or the high level of damage markers is compensated for by high levels of antioxidant protection [54]. After evaluating the obtained results for calculating the oxidative damage score in all three investigated mediums, DS was positively correlated with different doses of Cd, but a significant correlation was only observed with a higher Cd dose. Additionally, a significant correlation was detected in the Pb_150_ group. The parameters used to calculate antioxidative defenses had a lower trend in all investigated groups, and the overall protection score was lower than the control, but these changes were not significant. Overproduction of pro-oxidant versus antioxidative protection in the Cd_30_ group and Pb_150_ group resulted in a high OXY-score value, indicating higher oxidative damage in these two experimental groups. Observed oxidative stress scores are presented in Table 4.

### 3.5. Bioelements Levels in Tissues

Experimental groups treated with 15 mg Cd/kg b.w. had lower blood Cu levels in comparison to the controls (*p* < 0.001). After mixture treatment, blood Cu levels were in line with the control and Pb_150_ group, but higher than the Cd_15_ group (*p* < 0.001). On the other hand, the most prominent decrease of blood Zn level was observed after exposure to 30 mg Cd/kg b.w. in contrast to the control and Cd_15_ group (*p* < 0.05, *p* < 0.001, respectively). Following metal mixture treatment, blood Zn levels were lower compared to each of the individual metal treatment groups (*p* < 0.05).

Treatment with the higher Cd dose resulted in a significant elevation in liver Cu levels over control values (*p* < 0.01). Furthermore, both Pb-treated groups exhibited increased liver Cu levels, with the most prominent elevation after mixture treatment (*p* < 0.01). Levels of Zn in the liver were increased after all dose regimes, but Pb treatments produced the most pronounced one. Namely, in both Pb groups, we observed a significant elevation in Zn levels compared to control values (*p* < 0.001).

Both Cd groups (Cd_15_ and Cd_30_) had lower renal Zn levels compared to controls (*p* < 0.001). A significant decrease in renal Zn levels was also observed after Pb treatments compared to the control. Following administration of the mixture, renal Zn levels were higher than after single Cd treatment, but similar to the Pb_150_ group. Bioelement levels in the rat blood, liver, and kidney are presented in Table 5.

### 3.6. Histopathological Analysis

**Figure 5:**
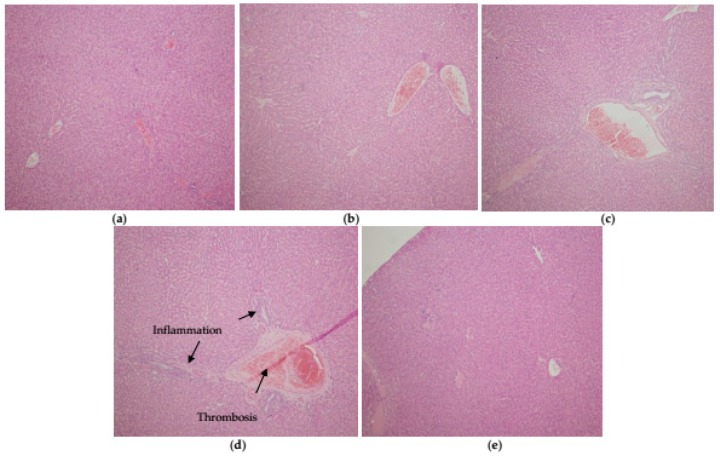
Effect of Cd or/and Pb on microstructures of rats liver after acute exposure. Panel (**a**): control group; panel (**b**): Cd_15_ group; panel (**c**): Cd_30_ group; panel (**d**): Pb_150_ group; panel (**e**): Cd_15_ + Pb_150_ group.

Histopathological examination in the liver of control rats showed normal architecture (Figure 5a). The Cd_15_ group cross-section showed very lightly dilated sinusoids with lymphocytic infiltrate in the portal spaces of the liver (Figure 5b), while lightly to moderately dilated sinusoids with mixed lymphocytic and neutrophilic infiltrate in the portal spaces were observed in the Cd_30_ group (Figure 5c). Lightly dilated sinusoids with mixed lymphocytic and neutrophilic infiltrate in the portal spaces, around biliary ducts, were observed in the Pb_150_ group. Additionally, scattered mononuclear cells in sinusoids were present. In zone 1 of the acinus, clear hepatocyte cytoplasm with condensed chromatin in nuclei was observed in the Pb_150_ group, while venous thrombus was present in only one of the examined tissues (Figure 5d). Treatment with the metal mixture resulted in a pattern of changes observed in the Pb treatment group, but the changes were more prominent (Figure 5e). The cross-section in the kidneys of the control group showed normal architecture, while in all treated groups, acute passive hyperemia without significant pathologic changes was observed.

## 4. Discussion

### 4.1. Effects on the Hematopoietic System

In general, the uptake, distribution, and accumulation of metals in the tissues and organs depend on many factors, such as the metals’ characteristics and forms, route, dose and exposure duration, the ability for binding to ligands in the cells, and species sensitivity. The hematopoietic system is one of the most sensitive organs to assess the toxicity. After oral administration, both Cd and Pb undergo intestinal absorption and are transported via blood. In the blood, they can be distributed via red blood cells and plasma proteins, mainly albumin [19,20]. Both Cd-treated groups had a significantly higher blood Cd concentration in comparison to the unexposed group, while there were no differences observed between Cd-treatment groups. It could be hypothesized that explanations for this occurrence are limited to gastrointestinal absorption, saturation of Cd binding sites in the blood, or fast clearance from the blood. On the other hand, analysis of toxic metals in the blood of experimental animals treated with a mixture showed some interesting trends. Namely, blood Cd concentration was lower than in the Cd_15_ group and it was in line with values we observed in the control group, while blood Pb concentration was higher than in the corresponding Pb group. Possible reasons for this observation might be the differences in their bioavailability and their competitive affinity to protein transporters following oral administration with possible antagonism involvement [55,56]. A similar observation was noted by Masso et al. [40] using dams that were consuming a solution of Cd (10 mg/L) and Pb (300 mg/L) from the first day of pregnancy and using the blood taken from pups at day 0 of parturition and the end of lactation. Co-exposure to these toxic metals resulted in diminished blood levels of both metals in contrast to single metal treatment, so the authors suggested a possible antagonistic effect due to gastrointestinal interactions.

Our data showed that both Cd doses led to absolute lymphopenia, with a more pronounced effect at the higher Cd dose, where lymphopenia was accompanied by leucopenia. Similar toxic effects of Cd on lymphocyte count were observed after single Cd treatment in rats [26,57], after 14 days of Cd administration in male BALB/c mice [58], and after four-week treatment of Wistar rats with CdCl_2_ via drinking water [28,59]. One possible cause of the leucopenia after a single Cd treatment in our study is that increased destruction of WBC is caused by Cd. We measured high levels of both metals in the spleen and thymus of treated animals after Cd administration (data are not shown). Lafuente et al. [60] showed high levels of Cd in the spleen and thymus after oral CdCl_2_ treatment with doses of 25–100 ppm, as well as decreased B lymphocytes levels in the spleen and thymus, suggesting that direct tissue toxicity is caused by Cd accumulation. Literature describing the Pb effect is conflicting, with some studies pointing to unchanged WBC status [25,61], leukocytosis [29], and leucopenia [62]. Neutrophilia with moderate leukocytosis observed in this study after mixture administration could be the result of neutrophil release and mobilization under inflammatory IL-6 and TNF-*α* from marginal neutrophil pools [63,64].

Administration of either of the toxic metals decreased RBC, HGB, and HCT, with the mixture group showing the largest effects. Namely, the percent decrease in RBC, HGB, and HCT ranged from 10 to 20% and was observed in both the Cd_15_ and Pb_150_ groups, with higher reductions observed in the Cd_15_ + Pb_150_ group (20 to 30%), corroborating possible additive effects of these metals. Our results are in agreement with other researchers using different animal models, route of exposure, and dose regimes, who also observed RBC, HGB, and HCT reductions [28,29,57,58,59,62,65]. We can assume that the reason for RBC, HGB, and HCT decreasing might be intravascular hemolysis since we measured high values of both metals in the rats’ blood in comparison to the control. Acute treatments of investigated toxic metals did not change MCV or MCHC, which is line with other authors [25,28,59]. Results from hemoglobin, MCV, and MCHC analysis indicated that both toxic metals may cause normocytic normochromic anemia, even after acute exposure. One of the possible mechanisms of metals-induced hemolysis is oxidative stress induction. Free radical production and lipid peroxidation induced by acute Cd treatment were implicated in RBC hemolysis and anemia in a study using Wistar rats treated by a single i.p. injection of 2 mg/kg b.w. CdCl_2_ [57]. Additionally, it is well-known that Pb binds to -SH groups of different proteins, including enzymes, and subsequently depresses the activity some of them, namely SOD and CAT [7,10,66] and produces a significant MDA and H_2_O_2_ increase in RBC accompanied with reduced glutathione levels [61]. The occurrence of early signs of oxidative stress in our study was observed after mixture treatment, through increased plasma LPO levels expressed as malondialdehyde (MDA). Furthermore, treatment with a higher Cd dose produced significant MDA elevation. These findings are consistent with our previous work, which revealed a significance increase of MDA levels in plasma after one Cd dose given orally (30 mg/kg b.w.) and i.p. (1.5 mg/kg b.w.) [42]. Apart from the lipid peroxidation of polyunsaturated membrane lipids, toxic metals can also produce oxidative damage of proteins, leading to AOPP generation, as shown in our previous study in rats [42]. In the present study, mixture treatment generated high plasma AOPP levels in contrast to all other experimental groups. Our results suggest that the effect of combined exposure to Cd and Pb resulted in a more pronounced negative effect in plasma versus individual metals, pointing to a possible additive effect. Total antioxidant status expressed as an additive effect of antioxidant molecules [48] was diminished in the blood of all treated groups, while TOS levels (including reactive oxygen metabolites, such as hydrogen peroxide and lipid hydroperoxides) were only elevated after single metal treatment with 30 mg/kg b.w Cd and 150 mg/kg b.w. Pb. In line with these results, OSI values were significantly elevated in plasma from the Cd_30_ group, as well as the Pb_150_ group, compared to the control. Toxic metal interference with the antioxidant system and resulting increased free radical load are reasons for the trend we observed in TAS and TOS values. Similar observations were noticed by Olisekodiaka et al. [67] and Buha et al. [42].

The two doses of Cd in the present work elicited contrasting results on platelets values. The lower dose of Cd (15 mg/kg b.w.) produced thrombocytosis in contrast to thrombocytopenia that was achieved with the higher Cd dose (30 mg/kg b.w.). The literature is somewhat conflicting, with some studies pointing to unchanged levels of PLT after acute or subacute treatment [26,68], and others showing a decrease in PLT account [28,57,59]. Thrombocytosis observed following administration of the lower Cd dose may be the result of inflammation, which is supported by the results from the WBC count, or as reactive thrombocytosis, which is transient and returns to normal after removal of the toxicant. Administration of the higher Cd dose or the single Pb dose resulted in thrombocytopenia. As we have previously mentioned, high concentrations of both metals were measured in the spleen (unpublished data), thus direct toxic effects of these metals on PLTs in the spleen represent a mechanism which explains the effects we observed. Interestingly, a mixture of Cd and Pb led to an attenuated effect of both metals given as single chemicals, which resulted in platelet values similar to the control group. In support of these findings, we have observed increasing spleen body mass in the Cd_30_ and Pb_150_ group, while it was not changed in the mixture group (data are not shown). Similar results with no changes in platelet count were observed in sub-chronic mixture studies cared out by Yuan et al. [24] and Cobbina et al. [25]. This finding suggests a possible antagonistic interaction of these two metals on the levels of PLT count.

### 4.2. Effects on Liver

In the present study, a significantly increased accumulation of Cd and Pb was observed in the liver. The concentration of Cd measured in the tissue correlated with the dose of Cd administered in that the 30 mg/kg b.w. Cd group had a Cd concentration in the liver that was twice as high as the 15 mg/kg b.w. dose. Measured levels of Cd in the liver were higher than in the blood or kidney, which was expected based on the route of administration and the duration of exposure used in the study. Additionally, the reason for this distribution is the high metallothionein (MT) synthesis in the liver [19,69]. Measured Cd concentration in the liver of the experimental group that received the mixture did not statistically differ from the individual dose regime, while Pb concentration was three times higher when compared to the single dose group. The obtained results are consistent with the measured levels of Cd and Pb in the blood after mixture administration (Cd level was in line with control group, while Pb level was higher than a single dose). Similar results were obtained in a fifteen-day study on rats i.p. treated with 0.05 mg Cd/kg b.w., 0.05 mg Pb/kg b.w., and a combined treatment of 0.025 mg Cd/kg b.w. + 0.025 mg Pb/kg b.w. [39], where after mixture treatment levels of Cd and Pb in the liver were in line with single dose regimes. A possible reason for this observation might be toxicokinetic interactions between these two metals on the level of intestinal absorption, and thereby modified distribution in blood and tissues. Divalent cation transporter (DCT1), also known as DMT1, is one of the carriers and a possible participant in the interaction between toxic metals [70].

Although the exact mechanisms involved in Cd and Pb hepatotoxicity are not fully understood, their ability to alter oxidative status is significant. In the present study, we observed high AOPP levels after single metal treatments accompanied with a TAS fall, while MDA levels were unchanged. The strongest TAS decrease was observed in the Pb_150_ group. Contrary to these results, Djukic-Cosic et al. [71], after 6h of a single Cd dose (20 mg/kg b.w), applied orally, observed MDA increasing in mice liver, but decreasing after 48 h, while Pillai et al. [39] reported an increase in TBARS levels after mixture and single Cd, but not after single Pb treatment administered i.p. 15 days. Moreover, no significant changes were observed in MDA and AOPP liver levels after mixture administration. Our results are consistent with results obtained by Cobbina et al. [25] and Masso et al. [40], who did not find any MDA/TBARS increase in the liver of animals treated with a Cd and Pb mixture. Despite the fact that Cd and Pb given individually induced some oxidative damage in the liver, the lack of the effects of Pb and Cd co-exposure may be at least partly explained by the role of MT and smaller proportion of free metal able to lead to the oxidative stress disturbance [7]. Metallothioneins exist in four major forms and are present in different tissues. Synthesis of metallothionein stimulates both investigated toxic metals, although Cd in a more profound manner. By binding to toxic metals, MT protects tissues from being affected by metal ions. The Cd-MT complex is primarily formed in the liver and then slowly released into the circulation and reaches the kidneys [7,19,56].

Changes in serum total protein values may indicate liver dysfunction as the liver is the main site of plasma proteins synthesis, primarily albumin [72]. Significantly lower values of total proteins and albumin were observed after mixture treatment, while single metals were not able to produce any change. Histopathological analysis did not reveal a serious disturbance of the liver structure that would lead to enzyme release. In line with this, we did not observe any elevation in serum AST and ALT activities. Contrary, both enzymes surprisingly showed a decreasing trend, but with significance only in the ALT activities of Cd_30_ and both Pb groups. While other authors recorded enzymes elevation [24,25,26], only a few record a decrease [73,74,75]. The observed decrease can be explained by the competition between toxic metals and divalent ions, such as Mg, Co, and Mn; activators of the enzyme; and Zn, a constituent of the enzyme [72]. This is supported by the fact that we measured lower serum values of Mg in all treated groups, with the most pronounced reduction in groups treated with Pb. Interaction between toxic metals and bioelements has been extensively studied by our research group and these studies confirmed many hypotheses; prolonged oral exposure to Cd significantly reduced Zn levels in blood and nine organs; Cd interferes with intestinal Mg absorption and affects its homeostasis; and Cd exposure results in negative effects on Mg, Zn, Cu, and even iron tissue levels [12,13,14,41,42,76,77].

### 4.3. Effects on Kidneys

In line with Cd doses, the experimental group that received 30 mg Cd/kg b.w. had a higher concentration in kidneys when compared to a lower Cd dose (73.5%). After mixture exposure, measured levels of toxic metals in the kidneys were higher than after single metal treatments. Possible explanations might be the excretory function of the kidney and rich blood supply and secondly, the role of the MT [2,78]. The formed Cd-MT complex is slowly released from the liver into the circulation and reaches the kidneys. After filtration in the kidneys, Cd is reabsorbed in proximal tubules and deposited in the kidneys. Metallothionein dissolves by discharging the free metals form capable of producing damage [7,19,56].

In the present work, higher Cd doses produced a more robust MDA elevation in kidneys compared to lower doses, yet these changes were not statistically significant compared to control values. On the other hand, Pb treatments increased kidneys MDA levels in comparison to the control, while the after mixture treatments increase was significant when compared to controls and the Cd_15_ group. Obtained results were similar to the results of other studies [28,29,59,62,79,80]. On the other hand, sub-chronic exposure to low Cd and Pb doses given as a single dose (0.005 mg Cd/L and 0.01 mg Pb/L), as well as a mixture through free drinking water [25], did not produce any changes in MDA levels in mice kidneys. The different study design, the path of exposure, the dose, as well as the applied chemical form of metals are some of the possible explanations for the different results obtained between researchers. As we have already mentioned, many researches have shown that both metals lead to the formation of oxidative stress, whether applied individually or together, with different doses and exposure pathways, as recently reviewed by Matović et al. [7]. Obtained results for MDA levels suggest that the effect of combined exposure to Cd and Pb resulted in a more pronounced negative effect in kidneys versus the effects of individual metals, which is contrary to the results obtained in the liver. The changes we observed correlate with the changes in measured Cd and Pb concentrations in the kidneys after administration of the metal mixture. Once again, the role and presence of MT could be important for these findings [7]. Additivity or synergism is a well-known scenario in co-exposure to Cd and Pb in kidneys. However, the limitation of our study is the fact that our study design is inadequate for interaction evaluation according to the criteria given by Borgert et al. [81], so we can only claim additivity.

The renal profile parameter, urea, significantly decreased in all experimental dosage regimes, compared to untreated animals, while creatinine levels exhibited an increasing trend. Changes in urea and creatinine levels indicate that even after the administration of a single dose of toxic metals, the excretory function of the kidney might be impaired. Similar results of changes in the levels of urea and creatinine were observed in other studies [24,25,26,28]. Impaired excretory function is also supported by the fact that levels of some serum ions have been changed (Ca, Mg, P, and Cl), as well as levels of Cu and Zn in the blood and kidneys. The strongest decreasing in serum levels of Ca and Mg was observed after mixture treatment in comparison to the control group. On the other hand, a single Cd dose (30 mg/kg b.w.) had a more prominent effect on the serum P level in blood, while a single Pb dose had a more pronounced effect on the Cl level. Additionally, a single dose of Cd (30 mg/kg b.w.) significantly decreased the Zn level in the blood, while all dose regimes decreased the kidney Zn levels.

## 5. Conclusions

Our results showed that acute exposure to Cd and/or Pb induced toxic effects in the blood, liver, and kidneys of adult Wistar rats. Oxidative stress is a major mechanism of toxicity for both metals and was implicated as a significant factor in our study, having observed a disturbed redox status in investigated tissues of treated rats. This study also showed a more profound toxicity of metal mixtures. Future studies should be focused on subacute or subchronic exposure to these metals and designed to enable interaction assessment.

## Figures and Tables

**Table 1:** Effect of Cd or/and Pb on hematological parameters in rats after acute exposure.

	Control Group	Cd_15_ Group	Cd_30_ Group	Pb_150_ Group	Cd_15_ + Pb_150_ Group
WBC (10^9^/L) ^1^	3.85 ± 0.68	3.56 ± 0.53	2.36 ± 0.32 *** ^††^	4.08 ± 0.75	3.97 ± 0.66
Lymphocyte (10^9^/L) ^2^	2.58 1.84–2.94	1.84 ** 1.36–2.11	1.15 ** ^††^ 1.01–1.16	2.54 2.18–2.77	2.22 1.44–2.69
Neutrophils (10^9^/L) ^2^	1.02 0.80–1.41	1.21 0.73–1.99	0.88 0.75–1.18	1.27 0.81–1.43	1.23 * 0.95–2.14
RBC (10^12^/L) ^1^	6.91 ± 0.25	5.61 ± 0.98 ***	5.05 ± 0.61 ***	5.74 ± 0.67 **	5.19 ± 0.74 ***
HGB (g/L) ^2^	148.0 143.0–161.0	139.0 * 105.0–154.0	117.5 *** 104.0–128.0	126.5 * 118.0–153.0	117.0 *** 94.0–127.0
HCT (L/L) ^2^	0.401 0.380–0.410	0.343 * 0.250–0.410	0.314 *** 0.260–0.340	0.336 * 0.300–0.420	0.303 *** 0.210–0.340
MCV (fL) ^2^	58.0 56.0–61.0	58.0 57.0–62.0	60.5 56.0–69.9	60.0 56.0–63.0	58.0 56.0–59.0
MCH (pg) ^2^	21.0 20.0–23.0	23.5 ** 22.0–25.0	23.0 * 22.0–26.0	22.5 22.0–24.0	22.0 21.0–26.0
MCHC (g/L) ^2^	370.0 361.0–390.0	398.0 379.0–420.0	386.5 349.0–409.0	383.0 361.0–397.0	379.0 372.0–447.0
PLT (10^9^/L) ^1^	571.25 ± 43.03	701.00 ± 40.30 **	341.50 ± 92.36 *** ^†††^	449.50 ± 100.60 **	525.00 ± 53.86 ^†††^

^1^ Values are presented as means ± standard deviation. ^2^ Values are presented as medians and ranges. WBC = white blood cell count; RBC = red blood cell count; HGB= hemoglobin; HCT = hematocrit; MCV = mean corpuscular volume; MCH = mean corpuscular hemoglobin; MCHC = mean corpuscular hemoglobin concentration; PLT = platelet count. Parameters that were not influenced by Cd and Pb treatment are not listed. Statistically significant differences (*p* < 0.05) compared to control group are indicated by *, ^†^ Cd_15_ group, ^‡^ Pb_150_ group. Statistical evaluation was performed using one-way ANOVA and Kruskal-Wallis nonparametric test followed by LSD and Mann-Whitney *U* test for pairwise comparison. * ^† ‡^ *p* < 0.05; ** ^†† ‡‡^ *p* < 0.01; *** ^††† ‡‡‡^ *p* < 0.001.

**Table 2:** Effect of Cd or/and Pb on biochemical parameters in rat serum after acute exposure.

	Control Group	Cd_15_ Group	Cd_30_ Group	Pb_150_ Group	Cd_15_ + Pb_150_ Group
BUN (mmol/L) ^1^	10.72 ± 1.02	8.56 ± 0.82 ***	8.76 ± 1.08 **	8.76 ± 1.12 **	9.08 ± 1.13 **
CRE (µmol/L) ^2^	42.45 39.5–43.4	44.75 35.5–53.4	45.45 ** 42.8–53.4	43.40 * 41.4–46.7	43.40 40.5–46.1
TP (g/L) ^2^	59.0 53.4–63.3	60.3 45.3–74.2	61.8 53.4–64.9	59.5 55.1–66.8	52.4 ** ^‡^ 48.7–58.0
ALB (g/L) ^2^	32.1 29.4–35.7	28.8 22.5–36.6	30.3 27.0–33.4	30.7 29.9–32.1	28.8 * 26.3–32.0
DB (µmol/L) ^2^	0.4 0.3–0.5	0.45 0.2–0.5	0.4 0.3–0.6	0.7 ** 0.7–0.9	0.4 ^‡‡^ 0.4–0.6
TB (µmol/L) ^1^	2.04 ± 0.16	2.56 ± 0.33 **	2.38 ± 0.38 *	2.24 ± 0.16	2.18 ± 0.14 ^†^
Fe (µmol/L) ^1^	61.7 ± 11.4	27.5 ± 8.8 ***	21.6 ± 5.8 ***	36.3 ± 10.5 ***	31.4 ± 6.3 ***
Ca (mmol/L) ^2^	2.93 2.5–3.2	2.73 2.2–3.2	2.65 2.5–3.1	2.65 * 2.5–2.9	2.48 ** 2.2–2.7
Mg (mmol/L) ^2^	1.70 1.5–1.9	1.55 1.1–2.2	1.30 * 1.3–1.8	1.35 *** 1.0–1.5	1.30 *** 1.1–1.5
P (mmol/L) ^2^	3.05 3.0–3.4	2.87 2.6–4.4	2.71 * 2.4–3.3	3.15 2.9–3.2	3.05 2.1–3.4
Cl (mmol/L) ^2^	105.0 102.0–109.0	110.5 102.0–118.0	108.5 * 105.0–116.0	113.0 *** 108.0–118.0	106.0 ^‡‡^ 100.0–109.0

^1^ Values are presented as means ± standard deviation. ^2^ Values are presented as medians and ranges. Blood urea nitrogen (BUN), creatinine (CRE), total serum proteins (TP), albumin (ALB), direct bilirubin (DB), total bilirubin (TB), iron (Fe), calcium (Ca), magnesium (Mg), inorganic phosphorus (P), chloride (Cl). Parameters that were not influenced by Cd and Pb treatment are not listed. Statistically significant differences (*p* < 0.05) compared to control group are indicated by *, ^†^ Cd_15_ group, ^‡^ Pb_150_ group. Statistical evaluation was performed using one-way ANOVA and Kruskal-Wallis nonparametric test followed by LSD and Mann-Whitney *U* test for pairwise comparison. * ^† ‡^ *p* < 0.05; ** ^†† ‡‡^ *p* < 0.01; *** ^††† ‡‡‡^ *p* < 0.001.

**Table 3:** Redox parameters in rat blood, liver, and kidney after Cd or/and Pb acute exposure.

		Control Group	Cd_15_ Group	Cd_30_ Group	Pb_150_ Group	Cd_15_ + Pb_150_ Group
Blood	MDA (µmol/L)	2.23 1.99–3.68	1.94 1.49–2.82	2.66 ^†^ 2.17–3.13	2.10 1.62–2.46	2.41 ^† ‡^ 2.16–3.21
	AOPP (µmol/g protein)	1.84 1.67–2.19	1.40 * 1.20–1.95	1.97 1.26–3.68	2.34 1.53–6.04	13.53 *** ^††† ‡‡‡^ 9.94–16.59
	SH-groups (mmol/L)	0.12 0.08–0.21	0.16 0.10–0.22	0.13 0.10–0.13	0.16 0.13–0.26	0.18 0.13–0.27
	PAB (HCU)	136.6 105.3–181.6	186.2 169.0–215.9	189.6 144.3–203.5	191.0 133.6–201.4	128.6 87.2–150.0
Liver	MDA (µmol/mg protein)	80.1 56.77–95.52	75.76 64.08–89.08	104.75 68.64–124.80	77.15 36.59–101.19	76.59 62.66–98.31
	AOPP (µmol/g protein)	83.16 35.82–166.36	234.84 *** 172.37–252.13	201.66 *** 179.94–324.36	178.99 * 114.81–228.23	81.42 ^††^ 38.05–194.69
	SH groups (mmol/g protein)	0.31 0.25–0.33	0.31 0.22–0.38	0.30 0.25–0.40	0.31 0.20–0.39	0.27 0.15–0.36
	SOD (U/g)	56.92 52.07–60.82	53.56 45.54–57.35	52.8 048.47–59.67	55.70 47.77–60.65	52.45 51.48–63.25
Kidneys	MDA (µmol/mg protein)	209.42 132.68–232.39	171.94 143.99–253.74	237.65 ^†^ 194.81–397.13	283.88 * 197.42–328.57	273.15 *** ^††^ 233.25–321.88
	AOPP (µmol/g protein)	273.68 153.97–341.22	255.58 206.36–317.21	286.65 250.53–360.61	297.75 231.56–368.82	273.79 226.38–362.08
	SH groups (mmol/g protein)	0.27 0.18–0.34	0.25 0.17–0.39	0.25 0.21–0.35	0.24 0.22–0.30	0.27 0.17–0.31
	SOD (U/g)	62.31 37.84–74.04	57.02 46.90–63.07	52.56 46.88–70.01	61.83 50.54–73.51	60.65 37.58–76.60

Values are presented as medians and ranges. Malondialdehyde (MDA), advanced oxidation protein products level (AOPP), total thiol (SH) groups level, prooxidative-antioxidative balance (PAB) expressed in arbitrary hydrogen peroxide complementary units (HCU), superoxide dismutase activity (SOD). Observed parameters are expressed on wet tissues. Parameters that were not influenced by Cd and Pb treatment are not listed. Statistically significant differences (*p* < 0.05) compared to control group are indicated by *, ^†^ Cd_15_ group, ^‡^ Pb_150_ group. Statistical evaluation was performed using the Kruskal-Wallis nonparametric test followed by Mann-Whitney *U* test for pairwise comparison. * ^† ‡^ *p* < 0.05; ** ^†† ‡‡^ *p* < 0.01; *** ^††† ‡‡‡^ *p* < 0.001.

**Table 4:** Oxidative stress score after acute exposure to Cd or/and Pb in rats.

	Control Group	Cd_15_ Group	Cd_30_ Group	Pb_150_ Group	Cd_15_ + Pb_150_ Group
Damage score	−0.083 ± 0.49	0.088 ± 0.51	0.526 ± 0.26 *	0.485 ± 0.24 *	−0.254 ± 0.39 ^‡^
Protection score	−0.038 ± 0.34	−0.37 ± 0.56	−0.29 ± 0.24	−0.11 ± 0.37	−0.22 ± 0.63
OXY-score	−0.045 ± 0.58	0.46 ± 0.89	0.81 ± 0.39 *	0.77 ± 0.30 *	−0.037 ± 0.80 ^‡^

Values are presented as means ± standard deviation. Statistically significant differences (*p* < 0.05) compared to control group are indicated by *, ^†^ Cd_15_ group, ^‡^ Pb_150_ group. Statistical evaluation was performed using one-way ANOVA followed by LSD post-hoc test for pairwise comparison.

**Table 5:** Levels of Cu and Zn in rat blood, liver, and kidney after acute exposure to Cd or/and Pb.

	Blood		Liver		Kidney	
	Cu (µmol/L)	Zn (µmol/L)	Cu (µmol/kg)	Zn (µmol/kg)	Cu (µmol/kg)	Zn (µmol/kg)
Control group	22.10 ± 1.81	87.76 ± 3.24	50.74 ± 2.80	462.77 ± 57.37	96.92 ± 10.39	374.18 ± 38.14
Cd_15_ group	16.09 ± 1.22 ***	93.19 ± 8.61	57.60 ± 5.58	585.16 ± 90.11 **	79.27 ± 4.06	299.70 ± 25.88 ***
Cd_30_ group	20.00 ± 2.34 ^††^	74.20 ± 15.81 * ^†††^	65.08 ± 10.48 **	576.49 ± 43.11 *	88.27 ± 21.02	310.47 ± 12.89 ***
Pb_150_ group	20.59 ± 2.40	92.23 ± 7.62	63.26 ± 3.65 *	613.06 ± 24.48 ***	110.95 ± 22.11	329.07 ± 22.95 **
Cd_15_ + Pb_150_ group	20.19 ± 1.53 ^†††^	81.04 ± 7.34 ^† ‡^	67.00 ± 13.17 **	609.78 ± 102.73 ***	95.42 ± 11.37	336.67 ± 24.44 * ^†^

Values are presented as means ± standard deviation. Levels of Cu and Zn are expressed as µmol/L in blood and as µmol/kg in liver and kidney (wet tissues). Statistically significant differences (*p* < 0.05) compared to control group are indicated by *, ^†^ Cd_15_ group, ^‡^ Pb_150_ group. Statistical evaluation was performed using one-way ANOVA followed by LSD post-hoc test for pairwise comparison. * ^† ‡^ *p* < 0.05; ** ^†† ‡‡^ *p* < 0.01; *** ^††† ‡‡‡^ *p* < 0.001.
